# Analgesic Effects of Paracetamol and Morphine After Elective Laparotomy Surgeries

**DOI:** 10.5812/aapm.12912

**Published:** 2014-03-08

**Authors:** Mahzad Alimian, Alireza Pournajafian, Alireza Kholdebarin, Mohammadreza Ghodraty, Faranak Rokhtabnak, Payman Yazdkhasti

**Affiliations:** 1 Department of Anesthesiology, Rasool Akram Medical Center, Iran University of Medical Sciences, Tehran, Iran; 2 Department of Anesthesiology and pain, Firoozgar Hospital, Iran University of Medical Sciences, Tehran, Iran

**Keywords:** Acetaminophen, Morphine, Laparotomy

## Abstract

**Background:**

Opioids have been traditionally used for postoperative pain control, but they have some unpleasant side effects such as respiratory depression or nausea. Some other analgesic drugs like non-steroidal anti-inflammatory drugs (NSAIDs) are also being used for pain management due to their fewer side effects.

**Objectives:**

The aim of our study was to compare the analgesic effects of paracetamol, an intravenous non-opioid analgesic and morphine infusion after elective laparotomy surgeries.

**Patients and Methods:**

This randomized clinical study was performed on 157 ASA (American Society of Anesthesiology) I-II patients, who were scheduled for elective laparotomy. These patients were managed by general anesthesia with TIVA technique in both groups and 150 patients were analyzed. Paracetamol (4 g/24 hours) in group 1 and morphine (20 mg/24 hours) in group 2 were administered by infusion pump after surgery. Postoperative pain evaluation was performed by visual analog scale (VAS) during several hours postoperatively. Meperidine was administered for patients complaining of pain with VAS > 3 and repeated if essential. Total doses of infused analgesics, were recorded following the surgery and compared. Analysis was performed on the basis of VAS findings and meperidine consumption.

**Results:**

There were no differences in demographic data between two groups. Significant difference in pain score was found between the two groups, in the first eight hours following operation (P value = 0.00), but not after 12 hours (P = 0.14) .The total dose of rescue drug (meperidine) and number of doses injected showed a meaningful difference between the two groups (P = 0.00). Also nausea, vomiting and itching showed a significant difference between the two groups and patients in morphine group, experienced higher levels of them.

**Conclusions:**

Paracetamol is not enough for postoperative pain relief in the first eight hour postoperatively, but it can reduce postoperative opioid need and is efficient enough for pain management as morphine after the first eight hours following surgery.

## 1. Background

Management of pain, especially postoperative pain, is a major concern for anesthetists in patients undergoing surgery. There are many ways for managing the postoperative pain. The most common way used after most surgeries is injecting analgesic drugs specially opioids. Excessive opioids administration is associated with a variety of side effects including ventilatory depression, drowsiness and sedation, nausea and vomiting, pruritus, ileus, urinary retention and constipation (1). Unpleasant side effects of opioids made investigators to search for some other analgesic drugs without these adverse effects. Non-steroidal anti-inflammatory drugs (NSAIDs) are another class of analgesics, used in some studies. Introduction of the newest short-acting analgesic drugs for intraoperative pain control and their widespread acceptance in anesthesia practice, has made the postoperative pain control a new dilemma to anesthesiologist, especially in more painful surgical procedures like laparotomy (2). Different classes of analgesics exert their effects through different mechanisms. NSAIDs' side effects like enteropathy may vary from drug to drug and be dose related (3). A combination of analgesics from different classes may provide additive analgesic effects with fewer side effects compared to a single therapeutic drug (3). Scientists are still seeking for new analgesic agents with fewer side effects. Paracetamol, intravenous form of acetaminophen, is a new compound which has been studied for postoperative pain control. There has been a trend over recent years for combining NSAIDs with paracetamol for management of the acute postoperative pain (4).

## 2. Objectives

The aim of our study was to compare the analgesic effects of paracetamol (as an analgesic that could be infused or injected intravenously) and morphine (as a traditional natural opioid) after elective laparotomy surgeries.

## 3. Patients and Methods

The study was approved by the legal and ethics committee of Iran University of Medical Sciences in March 2011 and registered at Iranian Registry Clinical Trials site (IRCT ID: IRCT201203114969N6). This double-blinded study which in, patients and evaluators were unaware of group assignments was performed on 159 ASA (American Society of Anesthesiology) class I-II patients aged between 25-85 years. Patients scheduled for elective laparotomy (with low midline incision in abdominal wall) were selected by block randomization in two groups. Patients with psychiatric illnesses, addiction, allergic reactions to opioids or paracetamol (or other NSAIDs) severe renal or hepatic disease and BMI ≥ 30 were excluded from the survey. Technique of general anesthesia was similar in both groups. After establishment of IV access and monitoring, midazolam (0.02 mg/kg) and fentanyl (2 µg /kg) was injected as premedication, and propofol (2 mg/kg) and atracurium (0.5 mg/kg) for Induction of anesthesia.

Propofol (100-150 µg/kg/min) as needed and remifentanil (0.4 µg/kg/min) were infused as maintenance and atracurium (0.15 mg/Kg) was injected every 30 minutes. Fifteen minutes before end of the surgery, fentanyl (1 µg /kg) was injected and at the end of the operation muscle relaxant effects was reversed by neostigmine (0.04 mg/kg) and atropine (0.02 mg/kg) .At the end of the surgery paracetamol, UniPharma, Greece (4 g/24 hours) or morphine sulphate, Daroupakhsh, Iran (20 mg/24 hours) injection would start through IV infusion pump. Visual analog scale (VAS) from 0 to 10 (with 0 representing no pain and 10 representing the worst imaginable pain) for postoperative pain was used in a time schedule (two, four, six, eight, 12 and 24 hours) after surgery.

For Any patient complaining of pain on with a score more that 3 on VAS 0.3 mg/kg of meperidine was injected and if demanded, the same dose was repeated until VAS ≤ 3. The total dose and number of doses injected was recorded. Postoperative nausea, vomiting, pruritus and respiratory rate were recorded throughout the study period. Data were registered in checklists including demographic characteristics (age, sex) and nausea, vomiting, pruritus, respiratory rate and urinary retention, patient’s complaints, visual analog scale score and meperidine dose were analyzed. Numerical variables were reported as mean ± standard deviation (SD). Quantitative and qualitative variables were measured by independent t-test, and ANOVA test respectively. P value ≤ 0.05 was considered to be statistically significant. All analyses were performed using SPSS for Windows version 19 (SPSS Inc., Chicago, IL, USA).

## 4. Results

Among all patients included in the study, 4 people in morphine group and five in paracetamol group were excluded due to different reasons and analysis was performed on the basis of VAS findings on the rest of the patients (n = 150, 75 in paracetamol group and 75 in morphine group). Mean age of patients was 54 ± 15.45 years. The data analysis showed both groups were similar regarding age, sex, BMI and duration of surgery (Table 1). The pain score in morphine group was lower than paracetamol group but had statistically significant difference in the first eight hours after operation (P value = 0.002), (Table 2). After 12 hours, despite lower scores in both groups, the difference was not meaningful (P value = 0.14). In both groups the VAS for pain intensity was lower than three, after eight hours (Table 3). The total dose of rescue drug (meperidine) and number of doses injected showed a significant difference between the two groups (P value = 0.004) (Table 4) .The cumulative doses of meperidine were significantly different in two groups (morphine versus paracetamol) over the study period. The nausea, vomiting and itching was lower in paracetamol group and showed a significant difference between two groups (Table 5). None of the patients experienced symptoms of respiratory depression during postoperative period. No late complications were reported.

**Table 1.** Demographic Data of the Patients (n = 75)

Characteristics	Morphine Group	Paracetamol Group
**Sex**		
Male	39	36
Female	36	39
**Age, y**	54.3 ± 16.7	53.7 ± 14.2
**BMI, kg/m^2^**	26.3 ± 3.32	27.1 ± 2.28
**Duration of surgery**	93.7 ± 12.28	96.9 ± 10.86

**Table 2.** Comparison of Pain Scores Between Two Groups ^a^

	Levine’s Test for Equality of Variances		T-test for Equality of Means		
	F	Sig.	Df	Sig. (2-tailed)	Mean ± SD
**Pain score after 2 hours**					
1	14.871	0	148	0	-0.76000 ± 0.10171
2			125.860	0	-0.76000 ± 0.10171
**Pain score after 4 hours**					
1	15.657	0	148	0	-0.90667 ± 0.10218
2			134.906	0	-0.90667 ± 0.10218
**Pain score after 6 hours**					
1	20.577	0	148	0	-0.61333 ± 0.15198
2			129.676	0	-0.61333 ± 0.15198
**Pain score after 8 hours**					
1	11.302	0.001	148	0.035	-0.25333 ± 0.11889
2			141.273	0.035	-0.25333 ± 0.11889
**Pain score after 12 hours**					
1	1.743	0.189	148	0.140	-0.13333 ± 0.08987
					
2			146.752	0.140	-0.13333 ± 0.08987
**Pain score after 24 hours**					
1	1.993	0.160	148	0.226	-0.12000 ± 0.09864
2			144.372	0.226	-0.12000 ± 0.09864

^a^ Abbreviations: Df, degree of freedom; f, Frequency; Sig, significance; SD: standard deviation.

**Table 3.** Mean Pain Scores in Two Groups

Group	Mean ± SD
**Pain score after 2 hours**	
Morphine	1.7333 ± 0.47458
Paracetamol	2.4933 ± 0.74204
Total	2.1133 ± 0.72849
**Pain score after 4 hours**	
Morphine	2.6933 ± 0.51918
Paracetamol	3.6000 ± 0.71660
Total	3.1467 ± 0.77188
**Pain score after 6 hours**	
Morphine	2.8000 ± 0.73521
Paracetamol	3.4133 ± 1.09166
Total	3.1067 ± 0.97724
**Pain score after 8 hours**	
Morphine	2.1333 ± 0.64375
Paracetamol	2.3867 ± 0.80360
Total	2.2600 ± 0.73667
**Pain score after 12 hours**	
Morphine	1.4267 ± 0.52436
Paracetamol	1.5600 ± 0.57516
Total	1.4933 ± 0.55256
**Pain score after 24 hours**	
Morphine	0.5200 ± 0.55410
Paracetamol	0.6400 ± 0.65016
Total	0.5800 ± 0.60501

**Table 4.** Mean Rescue Dose in Both Groups

Group	Mean ± SD	No.
**Meperidine dose after 4 hours**		
Morphine	0	75
Paracetamol	5.4667 ± 5.01170	75
Total	2.7333 ± 4.47164	150
**Meperidine dose after 6 hours**		
Morphine	0.5330 ± 0.0003	75
Paracetamol	5.2000 ± 5.02964	75
Total	2.6000 ± 4.40104	150
**Meperidine dose after 8 hours**		
Morphine	0	75
Paracetamol	0	75
Total	0	150
**Meperidine dose after 12 hours**		
Morphine	0	75
Paracetamol	0	75
Total	0	150
**Meperidine dose after 24 hours**		
Morphine	0	75
Paracetamol	0	75
Total	0	150

**Table 5.** Rate of Complications in Two Groups (n = 75) ^a^

Characteristics	Morphine Group	Paracetamol Group	P Value
**Nausea, vomiting**	41 (54.7)	26 (34.7)	0.034
**Itching**	29 (38.7)	1 (1.3)	0.003

^a^ Data are presented in No. (%).

## 5. Discussion

Effective postoperative pain control is essential for the optimal care of surgical patients. Actually, “pain relief is an essential human right” (5). NSAIDs and acetaminophen (paracetamol) are commonly used in the management of moderate to severe pain alone or in combination with opioids (6). Paracetamol is inhibitor of the synthesis of prostaglandins (PGs) and has some effects similar to those of the selective cyclooxygenase-2 (COX-2) inhibitors, *in vivo* (7). In the current study comparison of analgesic effect of paracetamol and morphine infusion after elective laparotomy surgeries were performed and the efficacy of paracetamol in pain killing after laparotomy was approved. Several studies show that paracetamol were commonly useful for postoperative pain control. Paracetamol behaves favorably according to the reduction observed in similar studies with different ketorolac (NSAIDs) doses, which were reported to produce a 31%-37% decrease in the morphine demand during the first 24 hours after surgery (8, 9). In some study no differences were observed between groups (paracetamol vs. placebo) in adequacy of analgesia, as assessed by VAS, although those values were only significantly lower at two intervals in the paracetamol group (10). The present study showed that although paracetamol (4 g in 24 hours) is not enough for postoperative pain relief, especially in first postoperative six hours, and patients needed rescue doses of meperidine, after eight hours the adequacy of analgesia was similar in two groups. This reduction in analgesic demand and decrease in the pain scores could contribute to a decrease in the side effects of using opioids alone. In some studies which evaluated analgesic drug combinations, results did not show a reduction of side effects, as might be expected due to a decrease in total morphine dose. This may be due to the limited number of patients included in these studies (11-16). Larger studies demonstrated the reduction of dose-dependent side effects of morphine, like sedation, respiratory depression, itching, nausea and vomiting (17). This study resulted in a significantly reduction of side effects of morphine, after paracetamol use. Another study by Gousheh et al. showed single use of paracetamol (1 g) had caused a better pain relief quality but it was not a suitable analgesic for moderate pain control in acute phase after surgery. In that study, patients undergoing laparoscopic cholecystectomy received paracetamol and placebo in different groups and found no significant difference in morphine consumption between the groups during the first six hours postoperatively (18). Mathiesen et al. compared adding paracetamol, pregabalin, dexamethasone and placebo postoperatively in three different groups and suggested that a combinations of paracetamol and pregabalin, or paracetamol, pregabalin and dexamethasone did not reduce morphine consumption and pain score compared to paracetamol alone, for patients undergoing abdominal hysterectomy (19). Paracetamol was used for postoperative analgesia in tonsillectomy patients and had more benefits in decreasing of bleeding versus rectal diclofenac (20). In another study, using 1 g of paracetamol as a single intravenous preemptive dose in abdominal surgery with perioperative epidural analgesia, did not reduce the consumption of the analgesics and the intensity of pain in the postoperative period (21). Paracetamol was also used as a pain killer in acute pains like renal colic (22). Some studies have been conducted for pain killing after the surgery and different types of drugs have been compared to each other (23-25) but paracetamol seems to be an effective and safe sole analgesic after laparotomy due to the lack of unpleasant side effects.

In conclusion, this study demonstrates the usefulness of paracetamol as an adjuvant to an opioid like morphine for treatment of postoperative pain after laparotomy surgery. Paracetamol infusion was associated with a satisfactory analgesia after eight hours, smaller opioid consumption and less adverse effects. The combination of intravenous form of acetaminophen (paracetamol) and morphine infusion may be beneficial in the management of acute pain after major surgery in patients, prone to opioid-related complications. Although, it is acceptable that paracetamol overall is an effective postoperative sole analgesic, it is recommend on the basis of this study findings, if it is used for pain killing after laparotomy surgeries, small amounts of opioids are essential for the first eight hours after operation.

Studying on various types laparotomy techniques was the limitation to this study; therefore it is better to repeat the study on specialized operations.
